# Binding of mycotoxins to proteins involved in neuronal plasticity: a combined *in silico*/wet investigation

**DOI:** 10.1038/s41598-017-15148-4

**Published:** 2017-11-09

**Authors:** Bernardina Scafuri, Antonio Varriale, Angelo Facchiano, Sabato D’Auria, Maria Elisabetta Raggi, Anna Marabotti

**Affiliations:** 1 CNR-ISA, National Research Council, Institute of Food Science, Via Roma 64, 83100 Avellino, Italy; 2 Scientific Institute, IRCCS “Eugenio Medea” Bosisio Parini, Via Don Luigi Monza 20, 23842 Bosisio Parini, LC Italy; 3 Department of Chemistry and Biology, “A. Zambelli”, University of Salerno, Via Giovanni Paolo II, 132, 84084 Fisciano, SA Italy

## Abstract

We have applied a combined computational procedure based on inverse and direct docking in order to identify putative protein targets of a panel of mycotoxins and xenobiotic compounds that can contaminate food and that are known to have several detrimental effects on human health. This procedure allowed us to identify a panel of human proteins as possible targets for aflatoxins, gliotoxin, ochratoxin A and deoxynivalenol. Steady-state fluorescence and microscale thermophoresis experiments allowed us to confirm the binding of some of these mycotoxins to acetylcholinesterase and X-linked neuroligin 4, two proteins involved in synapse activity and, particularly for the second protein, neuronal plasticity and development. Considering the possible involvement of X-linked neuroligin 4 in the etiopathogenesis of autism spectrum syndrome, this finding opens up a new avenue to explore the hypothetical role of these xenobiotic compounds in the onset of this pathology.

## Introduction

Mycotoxins are small molecules produced by the secondary metabolism of fungi or moulds that contaminate agriculture products and that exert toxic effects on humans. They are found worldwide in foods derived in particular from cereals, nuts, seed oils^1^, but also in foods of animal origin, such as milk and milk derivatives, eggs and meat, obtained by animals fed with contaminated fodder^2^. The presence of mycotoxins in food is monitored, and government agencies have established limits for the maximum allowed content of these compounds in food products. Nevertheless, their presence in foods such as cereals and cereal-derived products is very high in most parts of the world^3^, therefore, humans are constantly exposed to their toxic effects. Of particular concern is the presence of mycotoxins in baby foods, because foods for infants are largely cereal-based, often made of a single type of grain, and the diet in the months after breast- or bottle-feeding includes a very limited variety of foods. Therefore, the potential risk of exposure to mycotoxins in infants is higher than in adults^4^.

Several negative effects of mycotoxins on human health are well known: they are usually associated with carcinogenesis, teratogenesis, hepatotoxicity, nephrotoxicity, immunotoxicity and neurotoxicity^5^. In particular, aflatoxins are known to be potent carcinogens in all animal species where they are genotoxic. Their known target is DNA, to which they bind once metabolized in the liver by cytochrome P450 enzymes to a reactive epoxide form, producing a covalent adduct that leads to a carcinogenic lesion. Ochratoxin A is known for its nephrotoxicity and is recognized as possibly carcinogenic in humans. Moreover, it possesses genotoxic and immunotoxic activity. Given that its structure is similar to the amino acid phenylalanine, it interacts with a number of enzymes that recognize phenylalanine as a substrate; additionally, it causes mitochondrial damage and interferes with oxidative phosphorylation. Zearalenone and its reduced form zearalenol is able to induce endocrine effects because of its structural resemblance to 17-β-estradiol, and it is considered as a phytoestrogen. Deoxynivalenol can induce the so-called “ribotoxic stress response”, oxidative stress and apoptosis, and at the macroscopic level it is associated with the alteration of intestinal and immune functions. Moreover, it can promote emesis and anorexia. Finally, gliotoxin is a mycotoxin with the ability to induce apoptosis and prevent the activation of the NF-kB pathway. It also generates reactive oxygen species by interacting with the pro-apoptotic protein Bak and conjugates with proteins^1,5,6^.

While the action of the mycotoxins on peripheral organs and tissues is well documented, the data illustrating their effect on the nervous system are less abundant. Yet, the exposure to mycotoxin-producing molds and fungi is often associated with neurological abnormalities such as neurocognitive deficits, neuropathies and demyelinating processes^7^. The alteration of cholinergic and dopaminergic transmission in the adult rat brain caused by the chronic exposure to aflatoxin B1 was documented in studies more than 30 years ago^8–10^. The alteration of biogenic amine levels in some brain regions was reported in a mouse model exposed to an aflatoxin B1-rich diet, and this alteration was attributed to changes in the activity of some metabolizing enzymes^11^. More recently, studies have shown that aflatoxin B1 is able to induce several histopathological alterations in the cerebral cortex and hippocampus in a rat model^12^. Moreover, it has been shown that maternal exposure to aflatoxin B1 and to its metabolite aflatoxin M1 allows the transfer of these toxins to milk and causes alteration in hippocampal neurogenesis with suppression of cholinergic signals in the offsprings^13^.

For ochratoxin A, negative effects on the nervous system have been reported. In particular, the exposure to a chronic low dose of ochratoxin A reduces striatal dopamine and may be associated with the earlier onset of parkinsonism^14^.

For gliotoxin, few effects on the nervous system have been reported. It was found to induce apoptosis and to reduce the phagocytic capacity of astrocytes in a cell culture model of cerebral aspergillosis^15^, and caspase-dependent neurite degeneration in a cell line (SH-SY5Y) derived from differentiated human neuroblastoma; this latter toxic effect increased in the presence of glutathione^16,17^.

For deoxynivalenol, several recent studies have provided more details regarding its action on the brain. In particular, the emesis and the anorexia typically induced by this toxin seems to be related to a central effect that also involves altered levels of cytokine expression. Deoxynivalenol is also known to induce alterations in brain neurochemistry in several animal models. However, the precise molecular and cellular pathways triggering these adverse effects are unknown. Moreover, many studies have focused on the effects of acute deoxynivalenol intoxication, but the possible consequences of chronic deoxynivalenol consumption on the nervous system are still to be elucidated^18^.

Overall, it is clear that mycotoxins are ubiquitous xenobiotics, the effects of which on human health are not fully understood, and thus, potentially, they could be involved in many pathologies of uncertain etiology. Their negative effects could derive from the chronic exposure to low levels of these toxic compounds, and the diet has an important role since cereals appear to be the main carriers of these contaminants. Additionally, newborns and infants may be particulary subject to exposure and to developing negative effects. The body of evidence cited here prompted us to explore the possibility that mycotoxins could be involved in the etiopathogenesis of the autism spectrum disorder (ASD)^19^, a group of developmental disorders for which a number of factors have been implicated in its pathogenesis, including a direct genetic component, accounting for about 25% of cases^20^, but also epigenetic and environmental factors^21,22^. There is evidence that ASD patients suffer from recurrent gastrointestinal disorders that strongly correlate with the severity of their behavioral symptoms, and that changes in their gut microbiome may have an important role in the onset of this neurodevelopmental disease^23,24^. After performing an extensive analysis of the literature, we formulated a hypothesis to explain the sequence of causative events for the development of regressive ASD. We postulated that gastrointestinal disorders and gut dysbiosis can promote the so-called “leaky gut syndrome” allowing the production by the microbiota and/or absorption from contaminated food of neurotoxic compounds that could lead to the development of ASD^25^, in vulnerable and not yet immune-competent children. It is interesting to note that in the past few years many studies and anecdotal reports have pointed out the positive effects of the so-called “gluten-free-casein-free diet” that is based on the elimination of cereals, milk and their derivatives from the diet on the cognitive and behavioral aspects of ASD patients^26,27^. To date, there is no consensus in the scientific community about the real efficacy of this approach^28,29^. However, considering that cereals and milk are the food components with the highest probability of being contaminated by mycotoxins, it is possible that the positive effects attributed to the elimination of gluten and/or casein could be due to the elimination of these contaminants. The motivation for the discrepancies registered in the different studies about the effect of this diet could be that the presence of these contaminants may fluctuate in time and in quantity.

In a previous study in which we have investigated the role of mycotoxins in ASD etiogenesis, we identified an epigenetic mechanism by which ochratoxin A may trigger ASD, and may explain the typical male prevalence of this disease^30^. More recently, in a pilot study performed on a small group of ASD patients, unaffected siblings and unrelated controls, a significant association between the level of ochratoxin A in urine and serum and ASD was found, and the possible mechanism of action of ochratoxin A in the pathobiology of ASD has been further substantiated^31^. Furthermore, we have performed a wider study of the association between mycotoxins and other variables in autism, and our results have shown that ASD patients have more mycotoxins in their body fluids than controls^32^. This evidence corroborates the suspicion that mycotoxins are involved in ASD etiogenesis.

In order to investigate the mechanism of action by which mycotoxins may be involved in ASD etiogenesis, in the present study, we first adopted a computational approach in order to explore whether some mycotoxins interact directly with target proteins involved in neurodevelopmental processes. We then confirmed some of these interactions using steady-state fluorescence spectroscopy and Microscale Termophoresis (MST). Our results suggest that some mycotoxins can indeed bind proteins involved in the modulation of synaptic plasticity.

## Results

### Computational screening

#### Targets selection

For each selected mycotoxin, thousands of possible protein targets among all those available in PDB database^33^ were retrieved with the inverse docking approach. We obtained a list of 537 protein targets that involve human and non-human protein structures common to multiple mycotoxins. After the selection steps described in the Methods, we obtained a list of structures of human proteins expressed in brain and/or involved in neurological diseases that we considered the most interesting targets for the selected mycotoxins.

Among these targets, we focused our attention on human acetylcholinesterase (AChE), an enzyme that is essential for neurological transmission^34^. This enzyme belongs to the same structural family as neuroligins (NLGNs), a group of proteins located in the postsynaptic membrane that plays a fundamental role in synapse formation and plasticity^35^. Looking at the PDB database, we found a few structures of the human X-linked NLGN4 (NLGN4X) protein isolated or in complex with different neurexins. Therefore, after a careful analysis of their structural quality, we decided to add the structure of the isolated human NLGN4X (PDB: 3BE8)^36^ to the list of the proteins to be further evaluated, hypothesizing that the same mycotoxins interacting with AChE may also bind to NLGN4X. The final list of the structures of the human protein targets selected for further analysis is shown in Table 1.

**Table 1:** Final list of protein target structures.

**Protein Name**	**PDB Code**	**Putative Ligand By Idtarget Search**
Amine oxidase [flavin- containing] B (MAO B)	2BK3	Ochratoxin A, gliotoxin, beta-zearalanol, deoxynivalenol
D-amino-acid oxidase	2DU8	Gliotoxin, deoxynivalenol, ochratoxin A
Aldo-keto-reductase family 1 member C3	1S1P	Aflatoxin M1, aflatoxin B2, aflatoxicol, alpha-zearalanol, gliotoxin
Tankyrase-2	3MHJ	Aflatoxin B2, Aflatoxin M1, aflatoxin M2, Aflatoxicol deoxynivalenol
Acetylcholinesterase	1B41	Aflatoxin B1, aflatoxin B2, aflatoxicol, ochratoxin A, gliotoxin, deoxynivalenol
cAMP-specific 3′,5′-cyclic phosphodiesterase 4D	1XOQ	Ochratoxin A, gliotoxin
Beta-Secretase 1	1FKN	Ochratoxin A, gliotoxin
5′(3′)-deoxyribonucleotidase	1Q92	Aflatoxin B1, aflatoxin M2, alpha-zearalanol
Glutamate carboxypeptidase 2	3D7F	Ochratoxin A, gliotoxin
GMP reductase 2	2C6Q	Ochratoxin A, gliotoxin
Inositol monophosphatase	1IMB	Ochratoxin A, gliotoxin
Nicotinamide N-methyltransferase	2IIP	Ochratoxin A, alpha-zeraralanol, beta-zearalanol
Kynurenine-oxoglutarate transaminase 1	3FVS	Ochratoxin A, gliotoxin
Carnitine O-acetyltransferase	1NM8	Ochratoxin A, gliotoxin
*Neuroligin-4, X-linked* ^a^	*3BE8* ^a^	*Aflatoxin B1, aflatoxin B2, ochratoxin A, gliotoxin, deoxynivalenol* ^b^

^a^not found by idTarget search.

^b^putative target ligands are assumed the same as acetylcholinesterase.

#### Docking results

**Figure 1:**
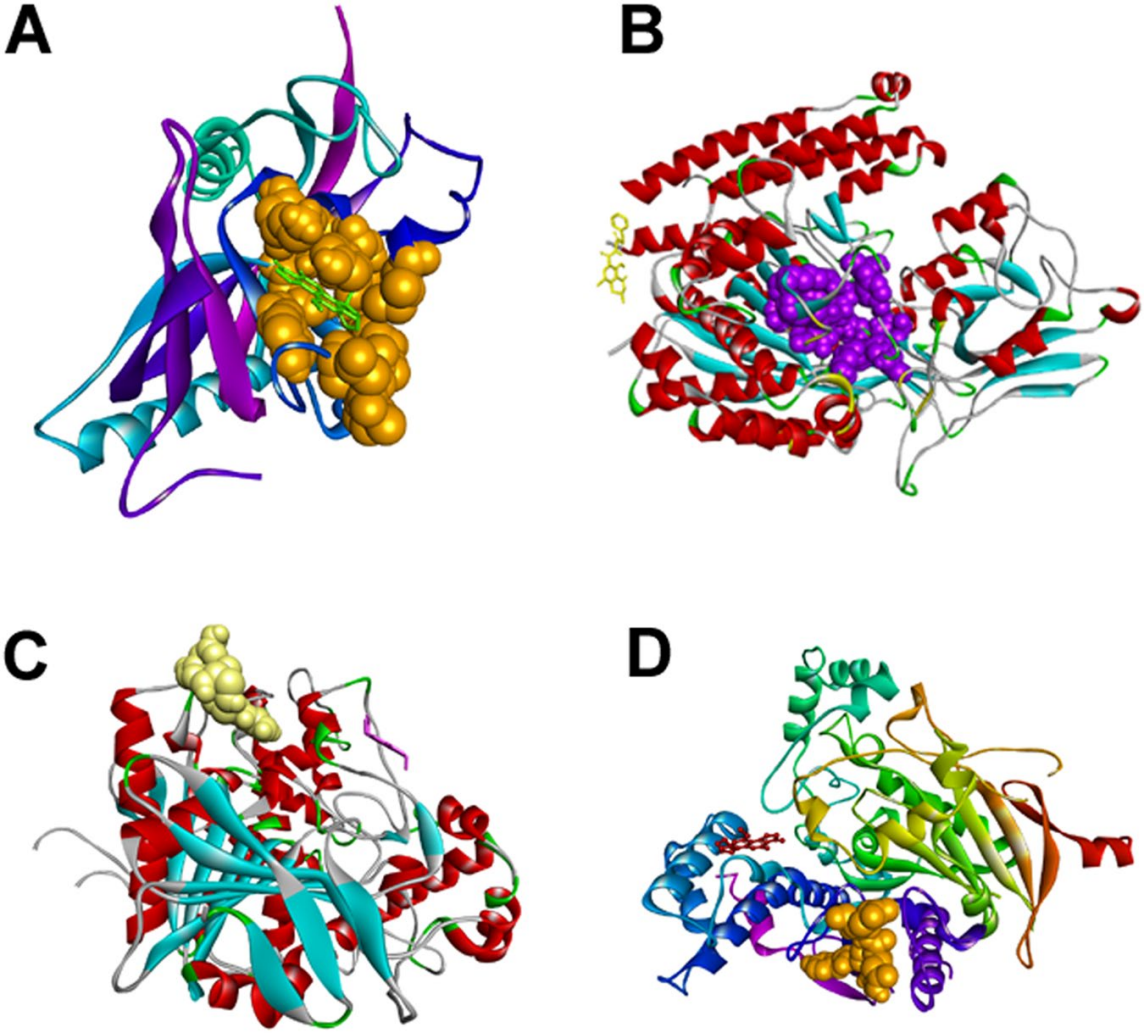
Results of blind docking between some mycotoxins and selected targets. (**A**) Aflatoxin M1 and tankyrase; (**B**) glutamate carboxypeptidase 2 and protonated ochratoxin; (**C**) aflatoxin B1 and AChE; (**D**) aflatoxin B2 and NLGN4X. The “canonical” binding site of the proteins is represented in spacefill mode, whereas the ligand is shown in stick mode. The picture has been created by Discovery Studio.

Blind docking results showed that some mycotoxins bind the selected protein targets in their “canonical” binding site with predicted binding energies varying between −11.04 kcal/mol to −4.27 kcal/mol (Supplementary Table 1). The best interaction predicted was the one between aflatoxin M1 and tankyrase 2 with a binding energy of −11.04 kcal/mol and more than 30 poses in the cluster represented by the selected pose (Fig. 1A), but other good interactions (predicted binding energy lower than −7 kcal/mol) were also found (see Supplementary Table 1). However, in most cases, we found that the selected mycotoxins could bind also cavities different from the active site, sometimes with a notable affinity. For example, ochratoxin A in its protonated form was predicted to bind glutamate carboxypeptidase 2 with an affinity of −9.27 kcal/mol in a site different from the canonical one, although in this case the cluster of results represented by the selected pose (Fig. 1B) was very small (only 2 poses).

Blind docking results for AChE showed that the best predicted interaction was the one with aflatoxin B2, with a predicted binding energy of −8.95 kcal/mol and a notable number of poses (56) in the cluster represented by the selected pose. However, also the other mycotoxins except deoxynivalenol can potentially bind the protein with a good binding energy and, especially in the case of gliotoxin, a high number of poses. In all cases but aflatoxin B1 (Fig. 1C), however, the predicted binding site was not the “canonical” one. Also in the case of NLGN4X, several mycotoxins were predicted to bind to the protein with a good energy. Aflatoxin B2 is the mycotoxin with the biggest cluster for the representative pose (Fig. 1D); aflatoxin B1 shows an even better binding energy and a notable cluster of poses. Also in this case, most mycotoxins were not predicted to bind to the canonical binding site as the preferred site of interaction (Supplementary Table 1).

**Figure 2:**
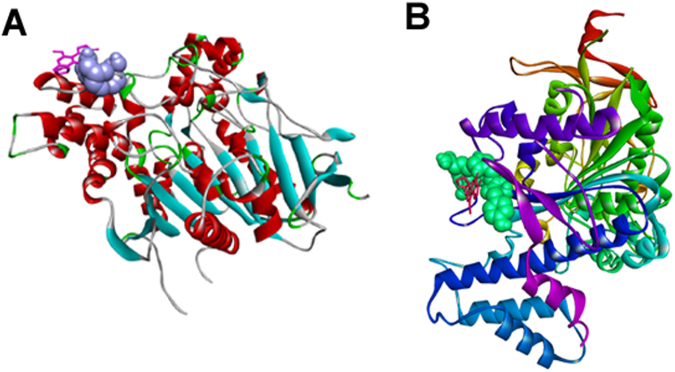
Results of focused docking for AChE and NLGN4X. (**A**) Aflatoxin B1 and AChE; (**B**) aflatoxin B2 and NLGN4X. The representation is as in Fig. 1.

In order to deepen our knowledge of the potential interactions between the mycotoxins and the two most interesting target proteins from our point of view, additional docking experiments focused on the canonical binding site were performed for AChE and NLGN4X, compared to the blind docking simulations. Results are shown in Supplementary Table 2. We found that the predicted binding energy for the interactions between AChE and the mycotoxins in the canonical binding site was significantly higher (worse) than the one predicted for the non-canonical binding site with the blind docking. However, in some cases, such as for aflatoxin B1 (Fig. 2A), the cluster of the representative solution was highly populated. On the contrary, the binding energy predicted for interactions between the canonical binding site of NLGN4X and the mycotoxins (Fig. 2B) was of the same order of magnitude as the one predicted with the blind docking. This allowed us to hypothesize that, for this target protein, the mycotoxins could bind to different binding sites having approximately the same affinity.

### Experimental validation

Unfortunately, among the list of different proteins identified as putative targets in Table 1, only AChE and NLGN4X were commercially available as recombinant human proteins with a degree of purity and in the appropriate soluble form suitable to perform the experiments. Moreover, only a very limited quantity of recombinant AChE was available from the producer; therefore, the quantity of assays performed with this protein has been necessarily limited. We validated the binding of some mycotoxins to these proteins by steady-state fluorescence spectroscopy and by MST assay.

#### Fluorescence steady-state results

The binding of the mycotoxins to the selected protein was estimated by a variation of their intrinsic tryptophan fluorescence emission. The number of Trp residues in NLGN4X and AChE is 20 and 13, respectively. They are located in several parts of the proteins and some of the predicted interactions with the mycotoxins do indeed occur near these residues (Supplementary Figure 1).

**Figure 3:**
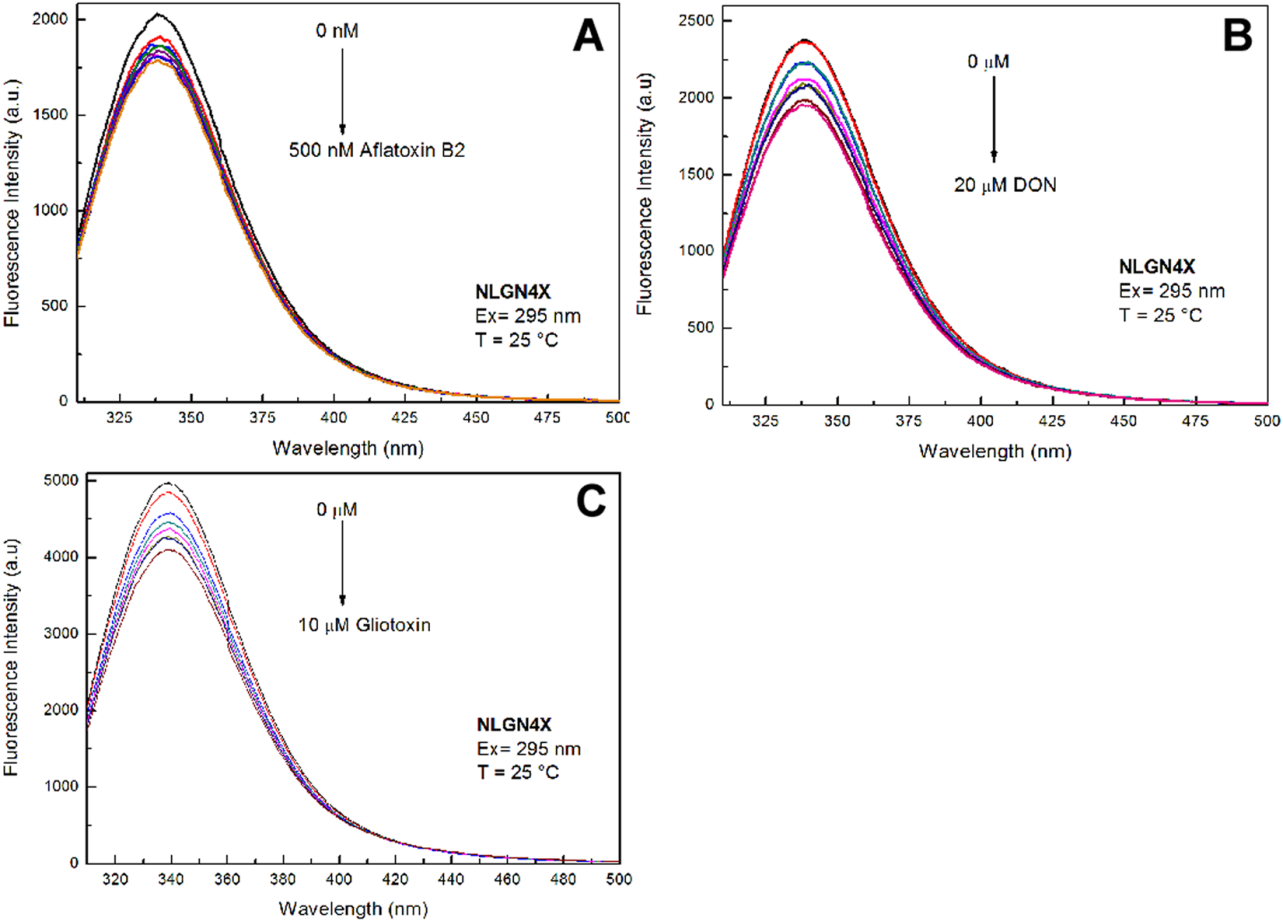
Fluorescence emission spectra of NLGN4X – aflatoxin B2 (**A**), NLGN4X – deoxynivalenol (**B**), NLGN4X – gliotoxin (**C**). The interaction of the mycotoxins with NLGN4X was registered by Trp fluorescence emission variation. All measurements were performed in PBS buffer pH 7.4 at 25 °C.

**Figure 4:**
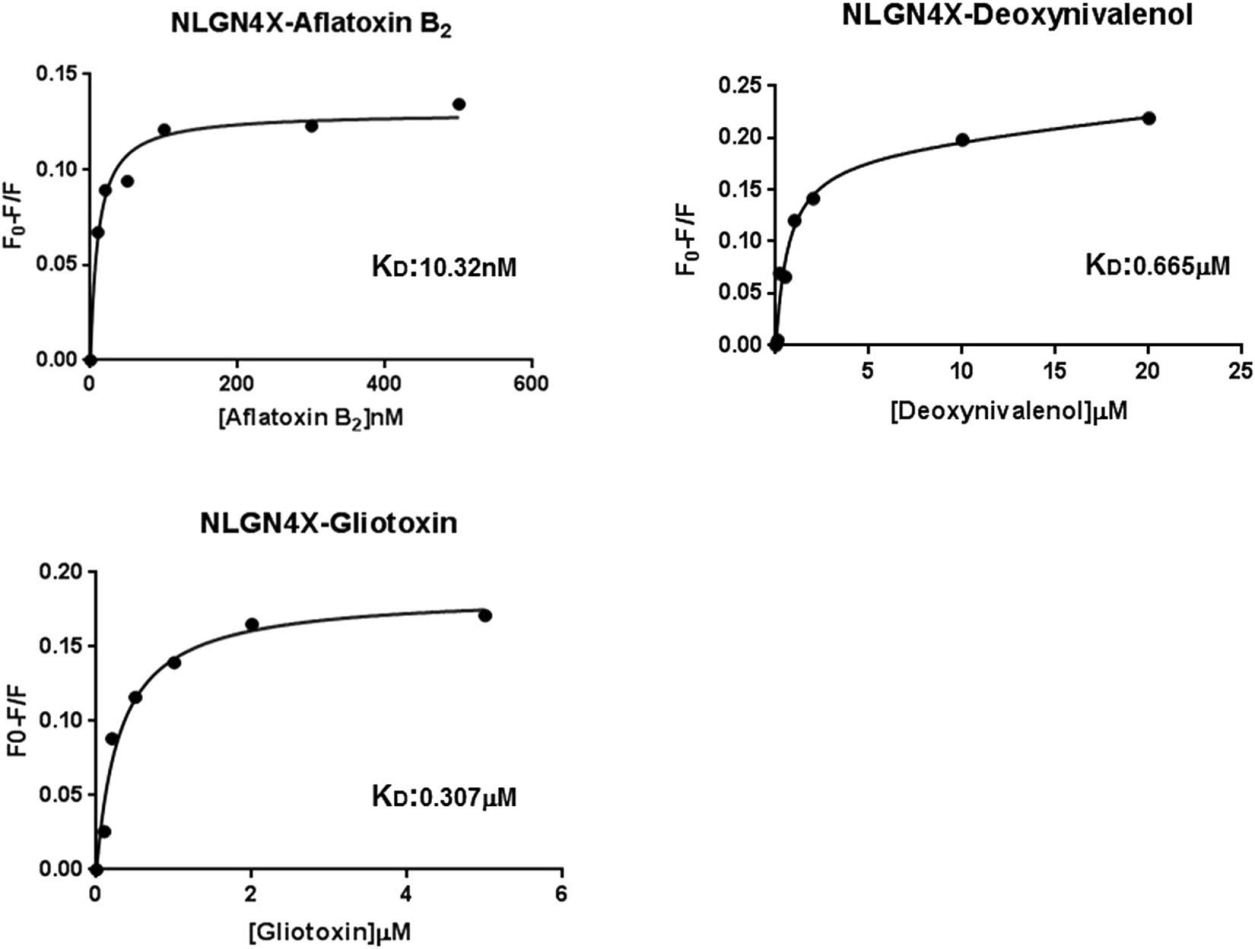
Interaction of NLGN4X with the selected ligand Aflatoxin B2 (**A**), deoxynivalenol (**B**) and gliotoxin (**C**). The curve shows the best theoretical fit to the analysed experimental data, R^2^ = 0.98.

NLGN4X binding experiments: Fluorescence steady-state experiments of NLGN4X were performed in the presence and absence of increasing concentrations of Aflatoxin B1 and B2, deoxynivalenol and gliotoxin. Figure 3A shows the fluorescence emission spectra of NLGN4X in the presence and absence of Aflatoxin B2 at 25 °C. The fluorescence emission spectrum of NLGN4X presents a peak centered at 337 nm. The position of the fluorescence emission maximum is blue-shifted with respect to the emission maximum of N-acetyl-tryptophanylamide (NATA) centered at 350 nm (data not shown), suggesting that the Trp residues of NLGN4X at 25 °C are in weakly buried and/or un-relaxed microenvironments. These data are in agreement with the position of the Trp residues in the protein structure (Supplementary Figure 1A). Fluorescence emission spectra show that, because of the addition of the mycotoxin, there is about 11% quenching of the protein fluorescence intensity. The non-linear regression analysis (Fig. 4A) of the fluorescence emission variation as a function of the toxin concentration showed that the dissociation constant (k_D_) calculated for the NLGN4X-Aflatoxin B2 complex is 10.32 nM. In Fig. 3B the variation of the fluorescence intensity of NLGN4X registered as a consequence of the addition of increasing concentrations of deoxynivalenol is reported. The addition of this molecule leads to a decrease of fluorescence emission of 17%. The k_D_ value calculated for this interaction was 665 nM (Fig. 4B). Figure 3C shows that the addition of gliotoxin to the protein leads to a decrease of emission fluorescence of about 17%, and the K_D_ value calculated with non-linear regression analysis was 307 nM (Fig. 4C). In contrast, the experiments performed with aflatoxin B1 did not show a significant decrease of the fluorescence emission spectrum at increasing concentrations of the toxin (data not shown).

The same sets of experiments were also performed in the presence of 0.5 M acrylamide. The obtained data still showed a quenching of the fluorescence upon ligand binding (data not shown). Together, these results suggest that there is an interaction between NLGN4X and three out of four toxins compounds tested (Aflatoxin B2, gliotoxin and deoxynivalenol), as predicted by the computational approach, whereas for Aflatoxin B1, the predicted binding with NLGN4X was not confirmed by this assay.

**Figure 5:**
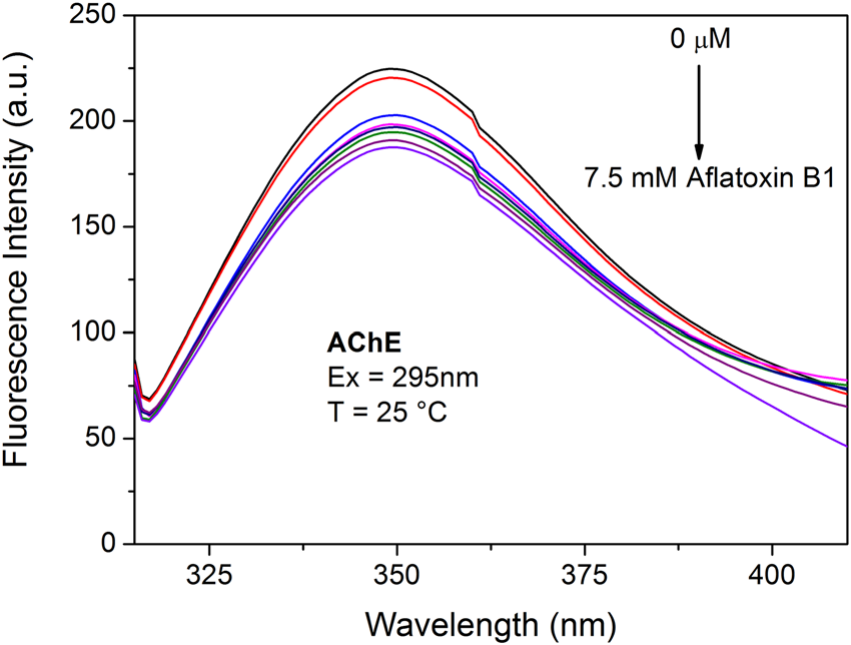
Interaction of the aflatoxin B1 with AChE measured by Trp fluorescence emission variation. The measurements was performed in PBS buffer pH 7.4 at 37 °C.

AChE binding experiments: Fluorescence steady-state experiments on AChE were performed in the presence and absence of increasing concentrations of aflatoxin B1 at 37 °C. The fluorescence emission spectrum of AChE (Fig. 5) presents a peak centred at 348 nm and the binding data show that, as a consequence of the increase of toxin concentration, a quenching effect was registered, suggesting the binding of the mycotoxin to the protein. However, the very limited quantity of the protein and its instability in the conditions of the assay did not allow us to calculate the k_D_ for this interaction.

#### Microscale thermophoresis (MST) results

**Figure 6:**
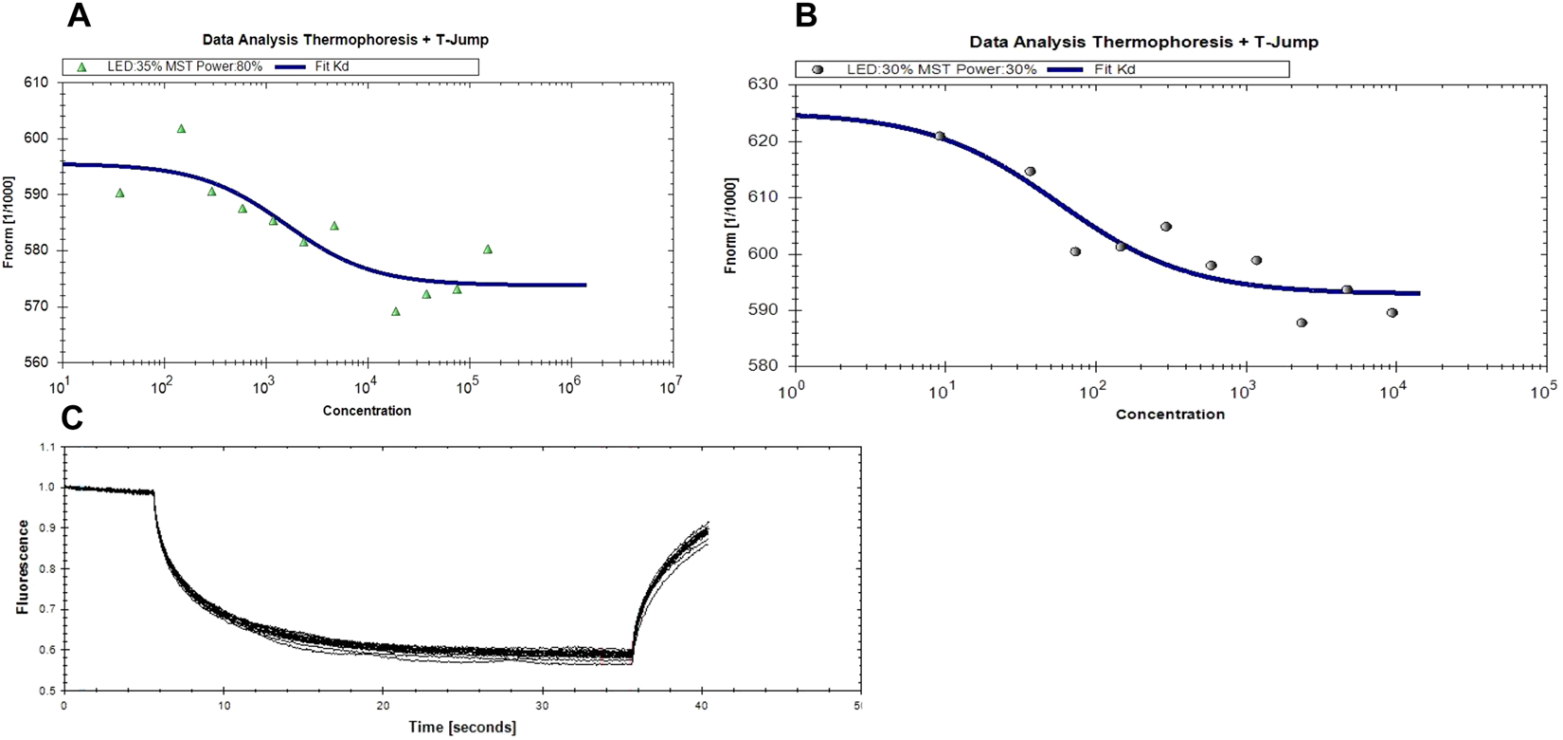
MST assay of NLGN4X with Gliotoxin (**A**), Aflatoxin B2 (**B**) and Aflatoxin B1 (**C**). All measurements were performed in PBS buffer pH 7.4 at 25 °C.

The interaction of NLGN4X with Aflatoxin B1, B2 and gliotoxin was further evaluated by MST assay. This was not possible for deoxynivalenol since this mycotoxin is not intrinsically fluorescent, as required by the conditions of this assay (see Methods). In Fig. 6A the MST assay data between NLGN4X and gliotoxin are shown. A k_D_ of 1.52 μM ± 254 nM was determined for this interaction, suggesting that there is binding between these two molecules. Figure 6B shows the data of the interaction between NLGN4X and Aflatoxin B2. The curve shows a biphasic shape suggesting the presence of two binding sites with different affinities. The k_D_ calculated from the curve for the binding site at higher affinity is 56.6 nM ± 7.38 nM. Figure 6C shows the results of the assay to test the interaction between NLGN4X and aflatoxin B1. These data show no evidence of the interaction between this mycotoxin and the protein, in agreement with the assay performed in steady-state fluorescence.

**Figure 7:**
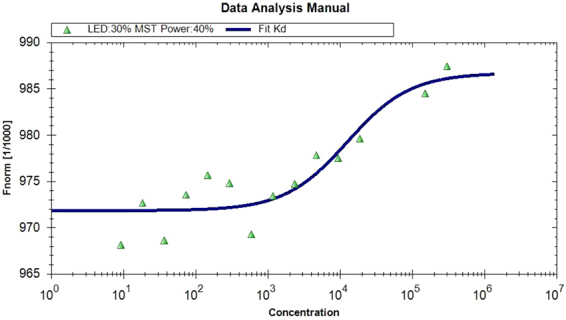
MST assay of AChE with Aflatoxin B1. The measurements were performed in Hepes buffer pH 8 at 25 °C.

MST experiments were also performed to test the interactions between AChE and selected mycotoxins. In Fig. 7 the binding curve obtained for Aflatoxin B1 is shown. The K_D_ calculated from this result is 8.4 ± 0.694 μM. This supports the data obtained by steady-state fluorescence (Fig. 5) suggesting the presence of an interaction between this mycotoxin and the protein. Assays were also performed to test the interaction between AChE and gliotoxin/Aflatoxin B2, but the results obtained did not confirm this prediction (data not shown).

## Discussion

Mycotoxins are well-known natural contaminants of foods derived from plants and animals fed with contaminated fodder, and probably the chronic exposure of humans to these compounds is greatly underestimated, despite the existence of restrictive regulatory limits for their intake. Many negative effects of these compounds are documented in the scientific literature, especially for peripheral organs; less is known about their effects on the nervous system, and the targets to which they can bind are not fully identified. In this study we tested whether some of these compounds can bind proteins involved in neurodevelopmental processes, using a computational approach. We then verified our computer predictions with a laboratory-based approach.

The results of the inverse docking approach suggested that many proteins are potential targets of these xenobiotic compounds. It is interesting to note that some of the targets found by this approach (Table 1) are involved in pathways known to be affected by mycotoxins, as deduced from information taken from specialized databases and online tools (see Methods). For example, aflatoxin B1 has been recently found to affect cholinergic transmission in rats and indeed we also found that AChE is a potential target for this mycotoxin. In addition, the identification of amine oxidase B, an enzyme involved in dopamine metabolism, as a potential target for ochratoxin A supports our predictions, since exposure to chronic low doses of this mycotoxin is associated with early parkinsonism^14^. Other target predicted by our approach can also elucidate toxic effects of mycotoxins, the targets of which are currently unknown, suggesting potential pathways that may be affected by the xenobiotic compound. We would like to point out that the list presented in Table 1 is only a small part of the targets found by the inverse docking approach, and includes only those human proteins that seem to be targets of multiple mycotoxins. The complete elucidation of all potential targets and pathways affected by mycotoxins is beyond the scope of the present article but it deserves to be studied. In the present study, we focused on the possibility that mycotoxins may be involved in ASD onset, and we pointed our attention on two enzymes involved in synaptic activity: AChE and NLGN4X. The first enzyme has been identified as a potential target for many different mycotoxins (see Table 1). AChE is a fundamental enzyme for neuronal transmission, since it terminates acetylcholine-mediated signal transduction at the neuromuscular junction by hydrolysis of this cationic neurotransmitter released into the synapse^34^. NLGNs are proteins that localize to the postsynaptic membrane where they are bound by neurexins (synaptic proteins located in the presynaptic membrane) and other proteins in both excitatory and inhibitory synapses^35^. There are several reports about the potential association of *NLGNs* genes and ASD; in particular, mutations of *NLGN3* and *NLGN4* genes, both located on the chromosome X, have been found in some ASD patients^37^. We decided to add the structure of NLGN4X to our analysis because its extracellular domain is structurally homologous to AChE and therefore potentially able to interact with the same mycotoxins as AChE. For these reasons, we focused on these two proteins in order to determine whether their predicted interactions with mycotoxins would be confirmed by other techniques, either computational or experimental. The direct docking approach suggested that NLGN4X can be bound by several mycotoxins (see Supplementary Tables 1 and 2) and that for both proteins the preferred binding site is not the catalytic site. When docking was forced towards the “canonical” binding site of NLGN4X, however, the predicted binding energy was still favourable and of the same order of magnitude as the alternative binding site. In contrast, for AChE, the predicted binding energy into the “canonical” binding site was significantly higher, suggesting that the active site of this enzyme is not the target of these xenobiotic compounds. This result suggests that, instead of blocking AChE activity, these compounds might modulate its activity e.g. in an allosteric fashion; however, further studies are needed to confirm this hypothesis.

We then used two different laboratory-based experimental approaches to verify the computer predictions. Steady-state fluorescence is a well-known and classical approach to test the binding of ligands to proteins. In particular, the variation of fluorescence intensity due to the tryptophan residues and a shift in wavelength of their emission maximum were chosen as indicators of protein conformational changes as a consequence of ligand interaction. In fact, due to its microenvironment dependence, tryptophan is an excellent intrinsic probe for the analysis of conformational changes of proteins upon binding of ligands^38^. In this study, this approach allowed us to confirm and characterize the binding of selected mycotoxins found by the computational approach to NLGN4X and AChE and calculate the kinetics parameters of the interactions.

MST is a new method that enables the quantitative analysis of molecular interactions in solution at the microliter scale. The technique is based on the thermophoresis of molecules, i.e. the direct motion of molecules in temperature gradients, which provides information about molecule size, charge, and hydration shell. Since at least one of these parameters is typically affected upon binding, the method can be used for the analysis of biomolecular interactions or modification of proteins or DNA. The MST uses an infrared laser for local heating and molecule mobility in the temperature gradient is analysed by fluorescence^39^. With this method, very small quantities of both protein and ligands are needed to perform the analysis. Thus, we were able to study the interactions between selected mycotoxins and AChE, for which only a very small quantity of protein was available. The MST analysis supported the results obtained with steady-state fluorescence for binding between aflatoxin B1 and AChE, but not for interactions with other mycotoxins. In contrast, for NLGN4X, MST analysis confirmed interaction with aflatoxin B2, thereby supporting the computational prediction of the presence of two possible binding sites with different affinities. In addition, the interaction with gliotoxin was confirmed, whereas the interaction with aflatoxin B1 was excluded, in agreement with steady-state fluorescence experiments.

In conclusion, our combined “*in silico*” and “wet” approach has highlighted the possibility that a direct interactions exist between mycotoxins and NLGN4X, a protein that is essential for synaptic plasticity. The role of synaptic dysfunction in neurodevelopmental disorders has been highlighted in the past^40^, and evidence in the literature supports the association between ASD and mutations in the *NLGN4X* gene^35^. Indeed, we have reported the presence of a statistically significant difference in mycotoxin levels between children affected by ASD and a control group in studies focused on the analysis of the exposure of children to these contaminants^31,32^. Moreover, in another study, we found several novel noncoding variants in the *NLGN3* and *NLGN4X* genes in the same cohort of patients and we discussed their association to ASD^41^. Our current results are another step towards a full elucidation of the putative role of natural xenobiotic compounds in ASD etiogenesis. In this study, we do not demonstrate the existence of such an involvement at the physiological level. More studies will be necessary in order to confirm if mycotoxins have a role in the onset of this pathology and, in particular, the physiological mechanisms by which such interactions may lead to the impairment of synaptic function and to the development of ASD. However, since this aspect is still essentially unexplored, we feel that our contribution opens the way for further analyses that may add an important piece to the jigsaw puzzle.

## Methods

### Computational procedures

#### Target selection

Twelve mycotoxin contaminants of milk and cereals (Aflatoxin B1, aflatoxin B2, aflatoxin M1, aflatoxin M2, aflatoxicol, ochratoxin A, patulin, deoxynivalenol, gliotoxin, α−zearalanol, β− zearalanol, zearalenone) were selected for this study. The files of their 3D structures (in .sdf format) were downloaded from the PubChem database (http://pubchem.ncbi.nlm.nih.gov)^42^. The structures of the mycotoxins were converted in the .pdb format using UCSF Chimera^43^. For ochratoxin A, bearing a carboxyl group, two different structures were designed in which this group was either protonated or deprotonated. An inverse docking approach was performed^44,45^ using idTarget (http://idtarget.rcas.sinica.edu.tw), a free web-server that identifies protein targets of small chemical molecules among the RCSB PDB database^31^ with robust scoring functions and divide-et-conquer docking approach^46^. The scanning mode has been used for the process; the other parameters were set to a default value.

We selected the first 100 protein targets obtained according to their best (lowest) predicted binding energy. In order to identify the most interesting targets, we focused on those proteins predicted to be target for many mycotoxins. In particular, we focused on human protein targets common to at least 2 mycotoxins, and on non-human protein targets common to 5 or more mycotoxins. For the latter targets, we searched for equivalent human homologues, if available, by performing a BLAST search^47^ among the protein structures stored in PDB database. The structural equivalence was further checked through visual inspection and superposition of the two structures.

For each selected protein target, the information about its molecular function and tissue specificity was retrieved from UniProt annotations^48^, and the molecular pathway and the disease(s) in which they are potentially involved were retrieved from KEGG database^49^. In this way, we selected those human protein targets that are expressed in the brain and/or involved directly or indirectly in neurologic diseases.

Finally, the structures of the selected targets were further checked for their reliability using the same procedure adopted by us in a previous work^45^, in order to select only those targets with the highest possible structural reliability for the following steps.

#### Molecular docking simulations

Molecular docking using AutoDock 4.2^50^ was performed to assess the interactions of the mycotoxins with the proteins selected by the previous steps. The molecular docking was performed by keeping the protein rigid, whereas the ligands were left flexible. Water molecules and other non-physiological ligands present in the crystallographic structures (cofactors excluded, when present) were removed from the .pdb file of the protein. For homodimeric proteins, the docking was performed on only one chain, by selecting the best one in terms of structural criteria. The ligands and the proteins were prepared using AutoDockTools^50^, by adding the hydrogens and partial charges according to Gasteiger^51^.

Two different docking approaches were performed: in a first step, the docking between the mycotoxins and their specific protein targets was performed by setting a grid box to include the entire protein surface. Subsequently, for the two proteins AChE and NLGN4X, a further docking study was performed with the grid box set to cover only the “canonical” binding site of the protein, as reported by the PDB file. In both cases, the dimensions of the grid box was set according to the protein’s dimension. The Lamarckian Genetic Algorithm was employed, setting 100 independent Genetic Algorithm runs for each ligand; the other parameters were kept at default value.

### Experimental procedures

#### Reagents

Human recombinant neuroligin 4-X linked (NLGN4X) was purchased from AcroBiosystem (Beijing, China), while human recombinant acetylcholinesterase (AChE) was obtained from Sigma Aldrich (Milano, Italy). The mycotoxins aflatoxin B1, aflatoxin B2, gliotoxin and deoxynivalenol were purchased from OrSell (Limidi di Soliera, Modena, Italy).

#### Fluorescence steady-state experiments

Fluorescence steady-state experiments were carried out on an FP-8600 Fluorescence Spectrometer (Jasco, Japan) equipped with a one-cell temperature-controlled sample holder. The binding of selected mycotoxins to the proteins was estimated by a variation of their intrinsic protein fluorescence emission. The stock solutions of aflatoxin B1, aflatoxin B2 and gliotoxin were prepared by dissolving the mycotoxins in DMSO at a concentration of 35.6 mM, 14 mM, and 30.6 mM respectively, whereas the stock solution of deoxynivalenol was prepared by dissolving this mycotoxin in distilled water at a concentration of 170 mM. The solutions of the selected proteins were prepared according to manufacturer specification: NLGN4X was prepared at a concentration of 1.25 μM in PBS at pH 7.4; AChE solution was prepared at a concentration of 0.17 μg/μl in HEPES 20 mM at pH 8.

For the protein solutions the excitation wavelength was fixed at 295 nm (maximum absorbance = 0.10 OD) and emission spectra were recorded between 310 and 410 nm with an emission slit width of 2.5 nm. Measurements were performed in PBS buffer at pH 7.4.

The variation of fluorescence intensity was registered from 0 to 50 μM for all assays, excluding the binding of Aflatoxin B1 to AChE (0 to 7.5 μM). The final protein concentration was adjusted by considering the final volume. The titrations made on AChE was performed at 37 °C (body temperature), but since NLGN4X is extremely unstable in those conditions, the temperature for the titrations on this protein was set at 25 °C. Experimental data were processed by a non-linear regression analysis computed with the Prism software (https://www.graphpad.com/scientific-software/prism/).

#### Microscale thermophoresis experiments

A Monolith NT.LabelFree (NanoTemper Technologies GmbH, Munich, Germany) was used for MST assays. In this assay, an intrinsic fluorescent probe is necessary to perform the assay, and the intrinsic fluorescence of the mycotoxins (excluding deoxynivalenol) was measured. To verify the interaction between AChE and Aflatoxin B1, the protein was prepared at a concentration of 100 nM in HEPES at pH 8.0, while to verify the interaction between NLGN4X, gliotoxin and aflatoxin B1 and B2, the protein was prepared at a concentration of 100 nM in PBS at pH 7.4. For each assay, 16 samples with 10 μl of mycotoxin solution at different final concentrations (from 300 nM to 9 nM) in DMSO 1% were prepared. 10 μl of protein solution was added to the 16 mycotoxins solutions. After a short incubation, the samples were filled into standard capillaries and the assay was performed following manufacturer’s indications.

### Data availability

Data generated or analysed during this study are included in this published article (and its Supplementary Information files). Data not included in the paper for size limits are available from the corresponding author on reasonable request.

## Electronic supplementary material

Supplementary Material
