# Supplementary material for: Binding of mycotoxins to proteins involved in neuronal plasticity: a combined in silico/wet investigation
Source: Sci Rep. 2017 Nov 9;7:15156. doi: 10.1038/s41598-017-15148-4 (PMC5680308; doi:10.1038/s41598-017-15148-4)

# SUPPLEMENTARY INFORMATION

# Binding of mycotoxins to proteins involved in neuronal plasticity: a combined *in silico*/wet investigation.

Bernardina Scafuri[1,2,3], Antonio Varriale[1], Angelo Facchiano[1], Sabato D'Auria[1], Maria Elisabetta Raggi[2], Anna Marabotti[2,3,*]

[1]: CNR-ISA, National Research Council, Institute of Food Science, Via Roma 64, 83100 Avellino, Italy

[2]: Scientific Institute, IRCCS "Eugenio Medea" Bosisio Parini, Via Don Luigi Monza 20, 23842 Bosisio Parini (LC), Italy

[3]: Department of Chemistry and Biology "A. Zambelli", University of Salerno, Via Giovanni Paolo II, 132, 84084 Fisciano (SA), Italy

*: Corresponding author. Phone: +39 089 969583; Fax: +39 089 969603; E-mail: amarabotti@unisa.it

Running title: binding of mycotoxins to proteins

**Supplementary Table 1.**

Results of the blind docking simulations for mycotoxins recognizing their selected human protein targets (see Table 1).

| LIGAND | BEST PREDICTED BINDING ENERGY (kcal/mol) | NUMBER OF POSES IN THE CLUSTER | CANONICAL BINDING SITE |
|---|---|---|---|
| **Amine oxidase [Flavin-containing] B (2BK3)** | | | |
| Ochratoxin A (deprotonated) | -10,43 | 3 | YES |
| Gliotoxin | -7,79<br>-7,08 | 3<br>38 | YES<br>NO |
| Beta-zearalanol | -7,39<br>-6,68 | 1<br>11 | NO<br>NO |
| Deoxynivalenol | -7,12<br>-6,99 | 1<br>32 | NO<br>NO |
| **Amino-acid oxidase (2DU8)** | | | |
| Ochratoxin A (deprotonated) | -8,05 | 7 | NO |
| Ochratoxin A (protonated) | -8,44 | 1 | NO |
| Deoxynivalenol | -5,04<br>-4,27 | 1<br>10 | NO<br>NO |
| Gliotoxin | -5,83<br>-5,25 | 5<br>12 | NO<br>NO |
| **Aldo-keto reductase family 1 member 3 (1S1P)** | | | |
| Aflatoxin M1 | -8,03 | 48 | NO |
| Aflatoxin B2 | -8,09 | 44 | YES |
| Aflatoxicol | -7,95 | 42 | YES |
| Alfa-zearalanol | -7,80<br>-6,95 | 2<br>21 | YES<br>YES |
| Gliotoxin | -8,23<br>-7,20 | 7<br>31 | YES<br>YES |
| **Tankyrase-2 (3MHJ)** | | | |
| Aflatoxin B2 | -10,77 | 38 | YES |
| Aflatoxin M1 | -11,04 | 32 | YES |
| Aflatoxin M2 | -9,93 | 26 | YES |
| Aflatoxicol | -10,41 | 25 | YES |
| Deoxynivalenol | -7,47<br>-6,32 | 3<br>13 | YES<br>NO |
| **Acetylcholinesterase (1B41)** | | | |
| Aflatoxin B1 | -8,37 | 58 | YES |
| Aflatoxin B2 | -8,95 | 56 | NO |
| Aflatoxicol | -8,82<br>-8,22 | 2<br>21 | NO<br>NO |
| Ochratoxin A (protonated) | -8,33<br>-7,81 | 8<br>11 | NO<br>NO |
| Gliotoxin | -7,45 | 71 | NO |
| Deoxynivalenol | -5,72 | 10 | NO |

| LIGAND | BEST PREDICTED BINDING ENERGY (kcal/mol) | NUMBER OF POSES IN THE CLUSTER | CANONICAL BINDING SITE |
|---|---|---|---|
| **cAMP-specif 3'-5'-cyclic phosphodiesterase 4D (1XOQ)** | | | |
| Ochratoxin A(deprotonated) | -7,62<br>-6,39 | 3<br>14 | YES<br>YES |
| Gliotoxin | -6,78 | 23 | YES |
| **Beta-secretase (1FKN)** | | | |
| Ochratoxin A (deprotonated) | -9,40<br>-9,13 | 19<br>27 | YES<br>YES |
| Gliotoxin | -6,62<br>-4,44 | 8<br>39 | NO<br>YES |
| **5(3) deoxyribonucleotidase (1Q92)** | | | |
| Aflatoxin B1 | -8,70 | 24 | YES |
| Alfa-zearalanol | -8,76<br>-8,37 | 17<br>20 | YES<br>YES |
| Aflatoxin M2 | -8,51<br>-8,41 | 23<br>24 | YES<br>YES |
| **Glutamate carboxypeptidase 2 (3D7F)** | | | |
| Ochratoxin A (protonated) | -9,27<br>-8,18 | 2<br>6 | NO<br>NO |
| Gliotoxin | -5,34 | 12 | NO |
| **GMP reductase 2 (2C6Q)** | | | |
| Ochratoxin A (protonated) | -7,10<br>-6,10 | 1<br>3 | YES<br>NO |
| Gliotoxin | -6,83 | 16 | YES |
| **Inositol monophosphatase (1IMB)** | | | |
| Ochratoxin A (protonated) | -7,05<br>-6,51 | 5<br>10 | NO<br>YES |
| Gliotoxin | -7,27<br>-6,58 | 4<br>27 | YES<br>NO |
| **Nicotinamide N-methyltransferase (2IIP)** | | | |
| Ochratoxin A (deprotonated) | -8,92 | 1 | NO |
| Alfa-zearalanol | -9,93<br>-7,16 | 4<br>37 | YES<br>NO |
| Beta-zearalanol | -7,12<br>-6,73 | 1<br>36 | NO<br>NO |
| **Kynurenine-oxoglutarate transaminase 1 (3FVS)** | | | |
| Ochratoxin A (deprotonated) | -5,56 | 8 | YES |
| Gliotoxin | -5,90 | 3 | YES |
| **Carnitine O-Acetyltransferase (1NM8)** | | | |
| Ochratoxin A (protonated) | -7,91 | 8 | NO |
| Gliotoxin | -6,78<br>-6,30 | 8<br>11 | NO<br>NO |

| LIGAND | BEST PREDICTED BINDING ENERGY (kcal/mol) | NUMBER OF POSES IN THE CLUSTER | CANONICAL BINDING SITE |
|---|---|---|---|
| **Neuroligin-4, X-linked (3BE8)[a]** | | | |
| Aflatoxin B1 | -7,17 | 27 | NO |
| Aflatoxin B2 | -6,94 | 36 | NO |
| Ochratoxin A (protonated) | -6,99 | 19 | NO |
| Ochratoxin A (deprotonated) | -7,49<br>-7,43 | 10<br>14 | YES<br>NO |
| Gliotoxin | -6,13<br>-5,43 | 5<br>13 | NO<br>NO |
| Deoxynivalenol | -5,05<br>-4,77 | 2<br>7 | NO<br>NO |

[a]: the docking were performed with the same ligands that recognize acetylcholinesterase in the inverse docking procedure

**Supplementary Table 2.**

Results of the docking simulations focused on the canonical binding site of AchE and NLGN4X for mycotoxins recognizing their selected human protein targets

| **Acetylcholinesterase (1B41)** | | |
|---|---|---|
| **LIGAND** | **BEST PREDICTED BINDING ENERGY (kcal/mol)** | **NUMBER OF POSES IN THE CLUSTER** |
| Aflatoxin B1 | -4,61 | 100 |
| Aflatoxin B2 | -4,13<br>-4,10 | 9<br>83 |
| Gliotoxin | -4,31<br>-4,02 | 40<br>59 |
| Aflatoxicol | -4,60 | 60 |
| Ochratoxin A (protonated) | -4,72<br>-3,34 | 8<br>26 |
| Deoxynivalenol | -4,12<br>-3,58 | 6<br>94 |
| **Neuroligin-4, X-linked (3BE8)** | | |
| Aflatoxin B1 | -7,06 | 51 |
| Aflatoxin B2 | -6.90<br>-6,49 | 20<br>62 |
| Gliotoxin | -6,77 | 74 |
| Deoxynivalenol | -6,58 | 89 |
| Ochratoxin A (protonated) | -7,19<br>-6,62 | 16<br>18 |
| Ochratoxin A (deprotonated) | -7,43 | 42 |

**Supplementary Figure 1: Position of the Trp residues in AChE (A) and NLGN4X (B).** The Trp residues are shown as sticks. The binding site of the mycotoxins is shown in CPK mode

A

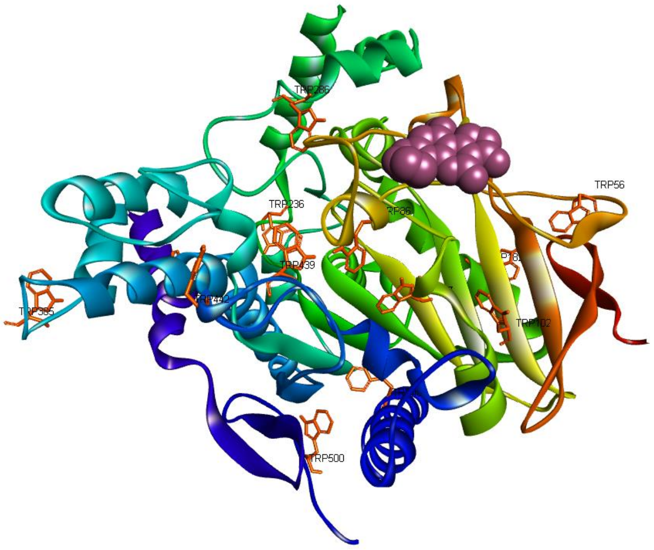


B

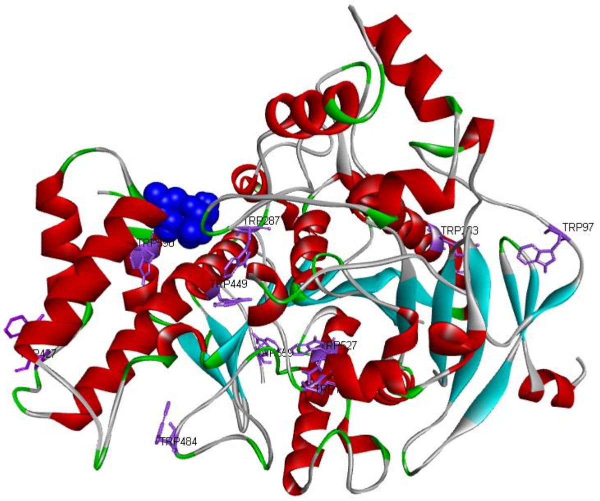

Supplement: Supplementary file 1 — Supplementary Material [file 41598_2017_15148_MOESM1_ESM.pdf]
